# Prediction Models of Mechanical Properties of Jute/PLA Composite Based on X-ray Computed Tomography

**DOI:** 10.3390/polym16010160

**Published:** 2024-01-04

**Authors:** Xintao Zhao, Junteng Li, Shangbin Su, Ning Jiang

**Affiliations:** 1 School of Transportation and Vehicle Engineering, Shandong University of Technology, Zibo 255000, China; zhaoxintaosdut@163.com; 2 School of Mechanical Engineering, Shandong University of Technology, Zibo 255000, China; 15053283217@163.com; 3 School of Metallurgy and Automotive Engineering, Shandong Vocational College of Industry, Zibo 255000, China; su_veh@126.com; 4 Shandong Ruicheng Aerospace Carbon Fiber Industrial Technology Institute Co., Ltd., Jining 272000, China

**Keywords:** jute/PLA composite, XCT, fiber orientation distribution, fiber length distribution, mechanical prediction model, tensile test

## Abstract

The tensile strength and modulus of elasticity of a jute/polylactic acid (PLA) composite were found to vary nonlinearly with the loading angle of the specimen through the tensile test. The variation in these properties was related to the fiber orientation distribution (FOD) and fiber length distribution (FLD). In order to study the effects of the FOD and FLD of short fibers on the mechanical properties and to better predict the mechanical properties of short-fiber composites, the true distribution of short fibers in the composite was accurately obtained using X-ray computed tomography (XCT), in which about 70% of the jute fibers were less than 300 μm in length and the fibers were mainly distributed along the direction of mold flow. The probability density functions of the FOD and FLD were obtained by further analyzing the XCT data. Strength and elastic modulus prediction models applicable to short-fiber-reinforced polymer (SFRP) composites were created by modifying the laminate theory and the rule of mixtures using the probability density functions of the FOD and FLD. The experimental measurements were in good agreement with the model predictions.

## 1. Introduction

Short-fiber-reinforced polymer (SFRP) composites are widely used in the automotive industry, commercial machinery, sporting goods, and the aerospace industry because of their low cost and easy processing [1,2,3]. SFRP composites are mostly processed using extrusion lamination and injection molding processes, which can increase productivity and reduce costs. In order to make full use of the mechanical properties of SFRP composites, the fibers should be oriented in the direction of the applied stress [4]. However, during the injection molding process, the fibers are not perfectly aligned in the composite. The vast majority of fibers are distributed in the mold flow direction, with some fibers being at an angle to the mold flow direction and having uneven fiber lengths [5,6,7,8,9,10,11]. The study of the mechanical properties of SFRP composites has become the subject of extensive research.

Due to the anisotropy of fibers, the mechanical properties of fibers in the longitudinal direction are superior to those in the transverse direction, resulting in the mechanical properties of reinforced composites being largely dependent on the fiber orientation [12,13,14]. Mortazavian [15] investigated the effects of fiber length, fiber orientation, and anisotropy on the elastic modulus and tensile strength of two short-glass-fiber-reinforced thermoplastics (polybutylene terephthalate with 30 wt% glass fibers, and polyamide-6 containing 35 wt% glass fibers and about 10 wt% rubber impact modifier) injection molded in the 0°, 18°, 45°, and 90° directions relative to the mold flow direction. Their experiments found that the tensile strength and modulus of elasticity of the composites both decreased significantly from fibers oriented in the mold flow direction to those in the direction perpendicular to the mold flow. Cordin [16] carried out tensile experiments on polypropylene-polyester fiber composites with different angles of fiber relative to the direction of modal flow. The results showed that the tensile strength and elastic modulus of the composites decreased with increasing orientation angle and that the mechanical properties of the composites were even lower than those of the matrix when the orientation angle was below a certain angle. Bernasconi et al. [17] studied the effect of fiber orientation on the fatigue strength of short-glass-fiber-reinforced polyamide-6, and conducted uniaxial tensile experiments and fatigue tests on tensile specimens relative to injection molding directions of 0°, 30°, 60°, and 90°. The experimental results indicated that the elastic modulus, ultimate tensile stress, and fatigue strength values decreased with the increase in the specimen orientation angle. Mohd et al. [18] investigated the mechanical properties of sugar-palm-yarn-reinforced unsaturated polyester resin composites with different fiber orientations. The results showed that composites with a 0° fiber orientation exhibited the maximum strength and modulus under tensile, flexural, compressive, and impact loads. The flexural strength of composites with a 90° fiber orientation was 85% lower than that of composites with a 0° fiber orientation. The above studies show that the mechanical properties of composites are related to the fiber orientation distribution and the mechanical properties of composites vary with the fiber orientation angle [19]. When the fiber orientation is consistent with the injection molding direction, the composites have the most desirable mechanical properties.

In the past decades, scholars have proposed some classical models to explain the effects of fiber length distribution (FLD) and fiber orientation distribution (FOD) on the mechanical properties of composites [20,21,22]. Kelly and Tyson [23] developed a tensile strength prediction model for steel fiber composites based on a plastic stress model and equilibrium for single fibers. Liang [24] proposed a tensile strength prediction model for short-fiber-reinforced polymer matrix composites considering interfacial influence factors. Halpin and Pagano [25] estimated the modulus of elasticity of randomly oriented short-fiber composites using the laminate theory. The theory considered several laminates of unidirectional short-fiber composites in different orientations. Fu and Lauke [26] extended the plane-only lamination analogy to three dimensions by combining the FLD and FOD probability density functions with the laminate theory to predict the elastic modulus of partially aligned short-fiber composites and found a good correlation between theoretical and experimental results. However, the above models were only rough estimations of fiber characteristics in the establishment process and were not suitable for direct application to SFRP composites. Therefore, an accurate quantitative analysis of the FOD and FLD of the short fibers in the composites is needed to modify the above laminate theory and rule of mixtures.

The mechanical properties of SFRP composites are closely related to the FLD and FOD [27]. In the past, the fiber length distribution has mostly been studied by direct measurement of fiber length in combustion resins [28,29,30], while the fiber orientation distribution has mostly been studied by optical methods such as microscopic imaging and confocal laser scanning [31,32,33,34], which are often destructive or do not extract sufficient fiber samples from the composites and cause errors. X-ray computed tomography has been applied as a non-destructive method to study the FLD and FOD of composite materials [35,36,37]. X-ray computed tomography is a non-destructive, high-resolution technique that allows the study of details within the material. The image obtained is essentially a three-dimensional map of the linear attenuation coefficients of the X-rays. It allows the three-dimensional visualization and quantitative analysis of the microstructure, such as the characteristics and spatial distribution of the fibers as well as their volume fraction. A reasonable prediction of the tensile strength and modulus of elasticity of SFRP composites is essential in order to ensure safe use and structural stability.

In this paper, the FLD and FOD of a jute/PLA composite were studied using X-ray computed tomography. The composites were reconstructed in three dimensions using the Avizo 2019 software, and the lengths and three-dimensional spatial coordinates (*θ*, *φ*) in the observation area (4 mm × 6 mm × 4 mm) were quantified. The effects of the FOD and FLD on the tensile strength and modulus of elasticity of the jute/PLA composite were investigated, comparing the mechanical properties of specimens at different loading angles (relative to the direction of mold flow). Analytical models were used to predict the tensile strength and modulus of elasticity of the composites. The experimental results were in good agreement with the predicted model.

## 2. Experiments

### 2.1. Experimental Materials

Polylactic acid (PLA) (4032D, molecular weight 150,000, density 1.25 g/cm^3^) was obtained from Suzhou Jiwang Environmental Materials Co., Ltd., (Suzhou, China). Jute fiber was obtained (three strands twisted) from Shanghai Qian Cong Jute fiber Co., Ltd., (Shanghai, China).

**Figure 1:**
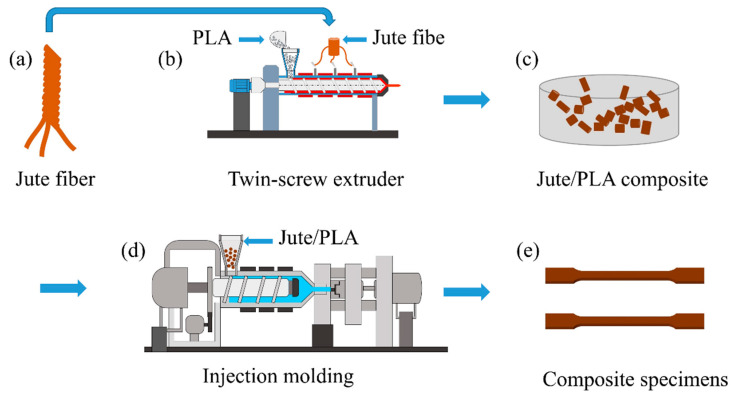
Process of short jute/PLA composite preparation: (**a**) Jute fiber; (**b**) Preparation of jute/PLA composite materials with 10% jute fiber content; (**c**) Cutting jute/PLA composite materials into particles; (**d**) Pour the dry composite particles into the injection molding machine; (**e**) Tensile specimens.

The jute/PLA composites were molded by extrusion injection molding, as shown in Figure 1. Jute yarn and PLA pellets were first vacuum-dried at 60 °C for 4 h. The jute/PLA composites with 10% jute fiber content were prepared using a twin-screw extruder (SHJ-20, Nanjing Jieya Co., Ltd., Nanjing, China) at 170 °C (Figure 1b). The jute/PLA composites were cut into pellets (Figure 1c) using a granulator (YCT-132, Nanjing Jieya Co., Ltd., Nanjing, China). The composite particles were dried under vacuum at 60 °C for 4 h. The dried composite particles were poured into an injection molding machine (PL550/150, Wuxi Haitian Machinery Co., Ltd., Wuxi, China) with an injection pressure of 55 bar and a barrel temperature of 170 °C (Figure 1d). The molten composite was injected all over the mold, and tensile specimens were obtained after cooling (Figure 1e).

### 2.2. Experimental Methods

#### 2.2.1. Tensile Test

**Figure 2:**
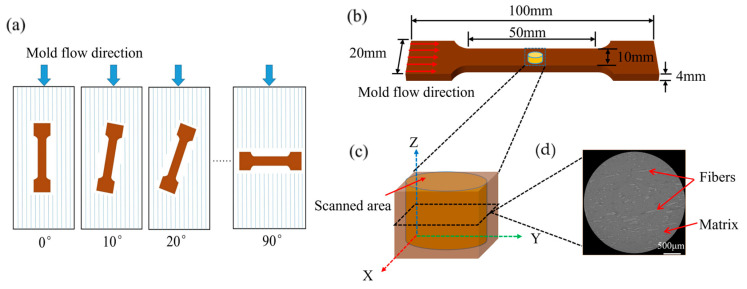
Tensile specimens and specimens for XCT: (**a**) five groups of loading angle specimens; (**b**) specimen size for tensile testing; (**c**) X-ray tomography observation area; (**d**) XCT image.

The tensile properties were tested according to the ATSM D3039 standard [38] using the mechanical testing machine WDW-20D (Jinan Hengsi Shengda Instruments Co., Ltd., Jinan, China). Five groups of specimens with different loading angles of 0°, 10°, 20°, 30°, and 40° were prepared (Figure 2a). Each tensile specimen in parallel cross-section had a length of 50 mm, a width of 10 mm, and a thickness of 4 mm (Figure 2b).

#### 2.2.2. X-ray Computed Tomography (XCT)

XCT tomograms of the fiber/PLA composite with a voxel size of 3 μm (medium resolution, MR) were obtained using an X-ray microscope (Xradia Versa 510, Zeiss, Oberkochen, Germany). Rectangular specimens with dimensions of 4 mm × 6 mm × 4 mm were taken from unstretched jute/PLA composite specimens. A detailed description of XCT was given in a previous article [39]. The specimens obtained from injection molding are shown in Figure 2a. The jute/PLA composites were examined using X-ray tomography, as shown in Figure 2c,d.

## 3. Results and Discussion

### 3.1. 3D Microanalysis

**Figure 3:**
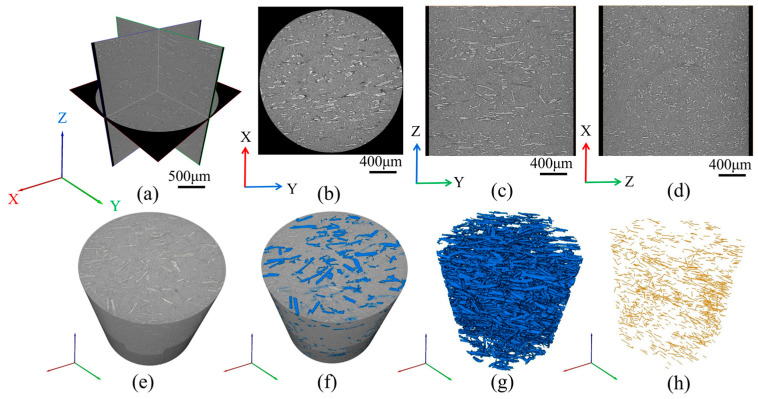
The jute/PLA 3D reconstruction process: (**a**) orthogonal images of the microstructure of the jute/PLA composite; (**b**–**d**) showing the fiber distribution in three sections; (**e**) 3D view; (**f**) threshold segmentation process; (**g**) separation of jute fibers; (**h**) jute fiber skeletonization process.

The 3D information of the jute/PLA composite was stored in 993 X-ray tomography images and the raw images of the scanned areas were imported into the Avizo 2019 software to obtain 3D visual images. Figure 3a shows a grayscale orthogonal view of the jute/PLA composite, where the lighter jute fiber and the darker PLA matrix can be more clearly distinguished. It can be seen from Figure 3b,c that most of the fibers are distributed along the Y-axis direction (i.e., the direction of mold flow) and from Figure 3d that most of the fibers are perpendicular to the XZ plane, also indicating that the fibers are distributed along the Y-axis. In order to obtain more accurate characteristics of the fiber orientation distribution, the fibers need to be separated and extracted. Firstly, the separation of fibers and matrix can be achieved using threshold segmentation, as in Figure 3f. Secondly, the segmented fibers are extracted, as shown in Figure 3g. Figure 3h shows the three-dimensional distribution of jute fibers after the skeletonization process. The fibers were found to be distributed mainly along the direction of the mold flow by observation.

**Figure 4:**
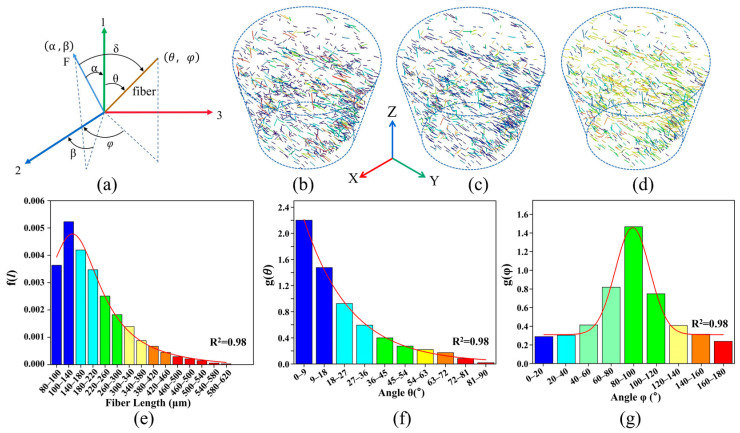
Quantitative analysis of jute fibers: (**a**) definition of fiber polar coordinates (*θ*, *φ*) and loading orientation angles (*α*, *β*); (**b**) fiber length distribution; (**c**) fiber *θ* orientation distribution; (**d**) fiber *φ* orientation distribution; (**e**) fiber length distribution variation; (**f**) fiber *θ* orientation distribution angle variation; (**g**) fiber *φ* orientation distribution angle variation.

The FLD and FOD of the skeletonized jute fiber were quantitatively analyzed using the Avizo software in order to represent the fiber flow direction more directly. The orientation of the jute fiber is expressed in Euler polar coordinates, i.e., polar angle *θ* and azimuth angle *φ*. The three-dimensional spatial coordinates of the jute fiber in the matrix are shown in Figure 4a, where F is the direction of load loading at coordinates (*α*, *β*). The coordinates of the jute fiber are (*θ*, *φ*). Axis 1 is the direction of the mold flow. *δ* is the angle between the loading direction and the fiber axis. The transformation relationship between the angle *δ* and (*θ*, *φ*) is given by Equation (1).

(1) cosδ=cosαcosθ+sinαsinθcos(φ−β)

In order to better quantify the 3D characteristics of jute fibers, the fiber length and fiber direction of the jute fibers were quantitatively analyzed using the XCT data. The smaller values are represented in blue, and the larger values are represented in red. The variation in jute fiber length is shown in Figure 4b,e, with an average fiber length of 220 μm and 70% of the jute fibers within the composite being less than 300 μm in length. The orientations of the jute fibers are expressed in Euler polar coordinates as polar angle *θ* and azimuthal angle *φ*, respectively, as shown in Figure 4c,f for the variation in polar angle *θ* of the fibers. The polar angle *θ* is less than 36° for nearly 75% of the jute fibers. As shown in Figure 4d,g, for the azimuthal variation in the fibers, nearly 70% of the fibers are predominantly distributed in the middle part of the composite (i.e., planes 1–3). This unique arrangement is due to the high shear stress of the material in the mold flow direction during the molding process. This distribution leads to anisotropy in the mechanical properties of the composite.

### 3.2. FLD and FOD Probability Density Functions

The 3D structure of the jute/PLA composite was reconstructed using Avizo and the diameters and angles of the jute fibers in the composite were measured. Figure 4e shows the fitted probability density function for the fiber length variation with an average fiber length of 220 µm. Equation (2) shows the fitted length probability density function:

(2) $$f(l)=\frac{3.16}{\pi }\times \left[\frac{231.6}{\left(4\times {\left(l-152.6\right)}^{2}+231\right)}\right]-0.000164$$

Figure 4f,g show the probability density functions fitted to the fiber orientation distribution, and Equations (3) and (4) give the fitted probability density functions for fiber orientation.

(3) $$g(\varphi )=0.31+1.15\times 2.72^{\left[-0.5\times {\left(\frac{\varphi -89.2}{15.4}\right)}^{2}\right]}$$

(4) $$g(\theta )=0.02038+3.38\times 0.06^{\theta }$$

### 3.3. Critical Fiber Length

**Figure 5:**
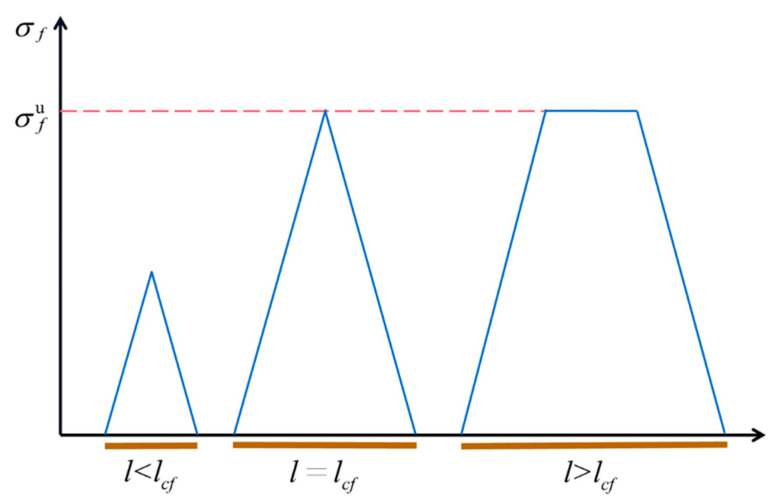
Variation in fiber axial stress with fiber length. The blue area represents the fiber axial stress and the red line represents the ultimate fiber strength.

The critical fiber length (*l_cf_*) is defined as the minimum fiber length required to bring the fiber axial force to the ultimate fiber strength (*σ_f_*) at the end of the fiber. The relationship between the fiber axial stress and the variation in fiber length is shown in Figure 5, and the critical fiber length is obtained using Equation (5) [40]:

(5) $$l_{cf}=r_{f}\frac{\sigma_{f}^{u}}{\tau }$$

where *r_f_* is the fiber radius and *τ* is the interfacial shear strength. The *τ* of short-jute-fiber-reinforced PLA is about 4–12.6 MPa [41,42,43], and the critical fiber length derived from Equation (5) is about 280 μm. Figure 4e shows that most fibers are less than the critical length.

### 3.4. Predictions of Elastic Modulus and Tensile Strength

#### 3.4.1. Prediction of Elastic Modulus

**Figure 6:**
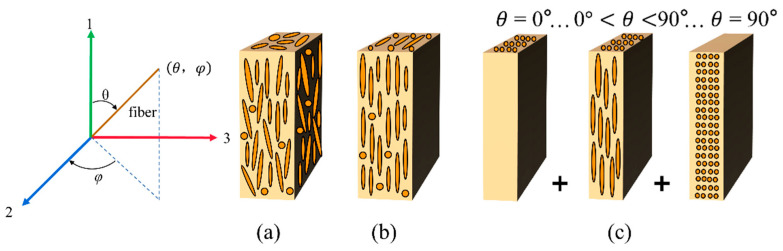
Simplified model of short-fiber composites: (**a**) the true form of short fibers in the matrix; (**b**) short-fiber composites at the same *g*(*θ*) and *φ* = 0; (**c**) laminate with the same fiber length and the same fiber direction.

The elastic modulus of short-fiber composites is related to the fiber length and the angle between the fiber spindle and the loading direction of the load, and the elastic modulus of the composites can be predicted by the theoretical model of the laminate, the basic idea of which is the use of a hypothetical composite superposition of planar-oriented composites in place of the actual composite material with three-dimensional spatial orientation, a simplified model of which is shown in Figure 6. It is assumed that the fiber morphology of SFRP is distributed in the matrix, as shown in Figure 6a. The partially aligned short-fiber composites are first replaced with hypothetical short-fiber composites having the same *g*(*θ*) and *φ* as a constant (0 ≤ *φ* ≤ π). When *φ* = 0 this hypothetical composite has no fibers passing through it from the XZ plane, as shown in Figure 6b. Based on the FLD and FOD of the fibers, the hypothetical short-fiber composite can be considered as a combination of multilayers, where each layer is considered to be composed of fibers of the same fiber length with the same fiber orientation, as in Figure 6c. Second, the overall stiffness constant of the simulated laminate is obtained by integrating the stiffness constant of each layer through the thickness of the laminate [37]. Finally, the modulus of elasticity of SFRP composites can be given by the relationship between the modulus of elasticity and stiffness constants of the laminate.

For computational convenience, the following assumptions are made: (1) the composite can be decomposed into an infinite number of laminates and the laminates are consecutive; (2) each laminate consists of fibers containing fiber length *l*~*l* + *dl*, polar angle *θ*~*θ* + *dθ*, and azimuthal angle *φ*~*φ* + *dφ*. For unidirectionally oriented composite laminates, take the fiber’s main axis direction as 1, in-plane perpendicular to the fiber’s main axis direction as 2, and perpendicular to the direction of the 1–2 plane as 3. The plane stress in the 3-axis direction of the stress component is zero. At this time, the stress–strain relationship of the single-layer plate is the positive-axis stiffness matrix, as shown in Equation (6).

(6) $$\left\{\begin{array}{l} \sigma_{1}\\ \sigma_{2}\\ \tau_{12} \end{array}\right\}=\left[\begin{array}{ccc} Q_{11} & Q_{12} & 0\\ Q_{21} & Q_{22} & Q_{26}\\ 0 & 0 & Q_{66} \end{array}\right]\left\{\begin{array}{l} \varepsilon_{1}\\ \varepsilon_{2}\\ \gamma_{12} \end{array}\right\}$$

where *Q_ij_* is the matrix of stiffness coefficients and the relationship between stiffness coefficients and engineering constants is shown in Equation (7):

(7) $$\left\{ \begin{array}{l} Q_{11}=\frac{E_{11}}{1-v_{1}v_{2}}\\ Q_{12}=v_{2}\frac{E_{11}}{1-v_{1}v_{2}}\\ Q_{22}=\frac{E_{22}}{1-v_{1}v_{2}}\\ Q_{66}=G_{12} \end{array}\right.$$

When the fiber orientation and the direction of the coordinate axis are not consistent, it is necessary to establish the stress–strain relationship under the off-axis coordinate system. The transformation equation between the on-axis system stiffness matrix components and the off-axis system stiffness matrix components ($Q_{ij}^{\prime }$) is

(8) $$\left\{\begin{array}{c} Q_{11}^{\prime }\\ \begin{array}{c} Q_{12}^{\prime }\\ Q_{22}^{\prime }\\ Q_{66}^{\prime } \end{array}\\ Q_{16}^{\prime }\\ Q_{26}^{\prime } \end{array}\right\}=\left\{\begin{array}{cccc} m^{4} & n^{4} & 2m^{2}n^{2} & 4m^{2}n^{2}\\ \begin{array}{c} n^{4}\\ m^{2}n^{2}\\ m^{2}n^{2} \end{array} & \begin{array}{c} m^{4}\\ m^{2}n^{2}\\ m^{2}n^{2} \end{array} & \begin{array}{c} 2m^{2}n^{2}\\ m^{4}+n^{4}\\ -2m^{2}n^{2} \end{array} & \begin{array}{c} 4m^{2}n^{2}\\ -4m^{2}n^{2}\\ {\left(m^{2}-n^{2}\right)}^{2} \end{array}\\ m^{3} & -mn^{3} & mn^{3}-nm^{3} & 2(mn^{3}-nm^{3})\\ mn^{3} & -nm^{3} & nm^{3}-mn^{3} & 2(nm^{3}-mn^{3}) \end{array}\right\}\left\{\begin{array}{c} Q_{11}\\ Q_{12}\\ Q_{22}\\ Q_{66} \end{array}\right\}$$

where *n* = sin *δ* and *m* = cos *δ*. The overall stiffness *A_ij_* is obtained by superposing the stiffness coefficients of all single-layer plates:

(9) $$A_{ij}=\sum_{k=1}^{M}Q_{ij}^{\prime }h_{k}$$

where *M* is the number of layers and *h_k_* is the thickness fraction of the *k*th layer. Thus, Equation (9) is written in integral form [44] as

(10) $$A_{ij}=\int_{\varphi =\varphi_{\min}}^{\varphi_{\max}}\int_{\theta =\theta_{\min}}^{\theta_{\max}}\int_{l=l_{\min}}^{l_{\max}}Q_{ij}^{\prime }f(l)g(\theta )g(\varphi )dld\theta d\varphi$$

Finally, the modulus of elasticity of the jute/PLA composite in any direction (*α*, *β*) can be obtained from Equation (11) [32]:

(11) $$E_{c}(\alpha ,\beta )=\frac{A_{11}A_{22}-A_{12}^{2}}{A_{22}}$$

**Figure 7:**
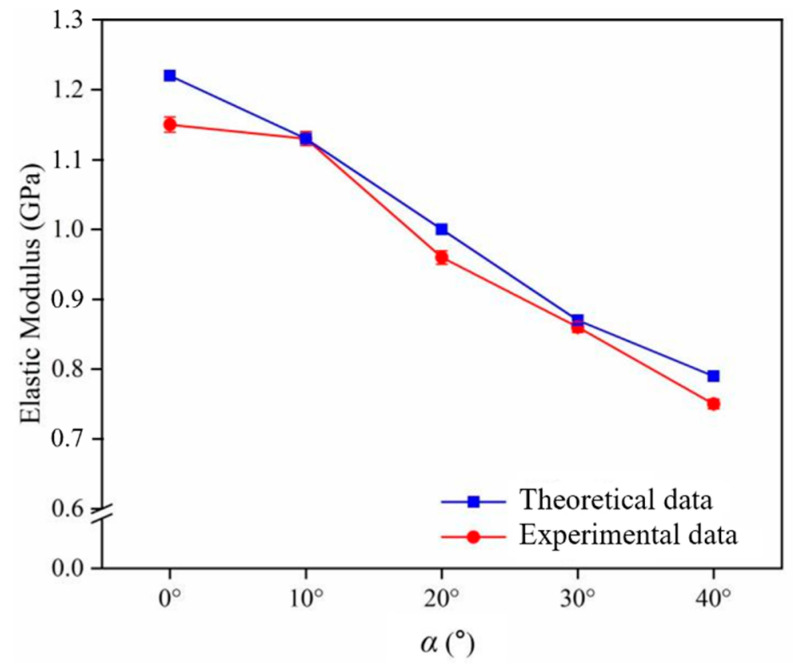
Variation in the modulus of elasticity of the composite with the angle *α* in the direction of force: comparison of experimental and theoretical values.

Validation of the theoretical model of tensile strength constructed follows. As the maximum angle of *δ* is 51.34°, the maximum value of *α* is obtained from Equation (1) as 40° when *β* is a constant value of 90°. The modulus of elasticity of five groups of composites were obtained from tensile experiments. The comparison of the theoretical and experimental values of the modulus of elasticity of the short jute/PLA composite is shown in Figure 7. The experimental values are close to the theoretical values. A comparison of the experimental and theoretical values shows that the modulus of elasticity of the short jute/PLA composite decreases as the loading angle increases. When the loading angle was 0°, the elastic modulus of the composite was 1.15 GPa, which was similar to the value measured by Karthika [45]. When the loading angle was close to 40°, the modulus of elasticity of the composite was 0.75 GPa, which was less than the modulus of elasticity of the matrix, of 0.85 GPa, indicating that the short jute fibers no longer played a reinforcing role. The above analysis of the XCT data visualization of the jute/PLA composite shows that this is due to the unique fiber arrangement of the jute fibers within the composite. The maximum relative error between the theoretical and experimental values was 6.1%. These errors are due to the fact that the proposed mechanical prediction model does not take into account the existence of defects at the interface between the fibers and the matrix or the varying diameters of the fibers, and are within acceptable limits, indicating that the proposed mechanical model is able to predict the modulus of elasticity of the short jute/PLA composite at any loading angle.

#### 3.4.2. Prediction of Tensile Strength

To establish the theoretical model of tensile strength of short-fiber composites, it is first necessary to analyze the single-fiber bridging stress across the fracture section according to the critical fiber length of partially oriented short-fiber composites, and then use the fiber probability density function to modify the mixing rate model to obtain the theoretical model of tensile strength of partially oriented short-fiber composites based on the single-fiber bridging stress.

**Figure 8:**
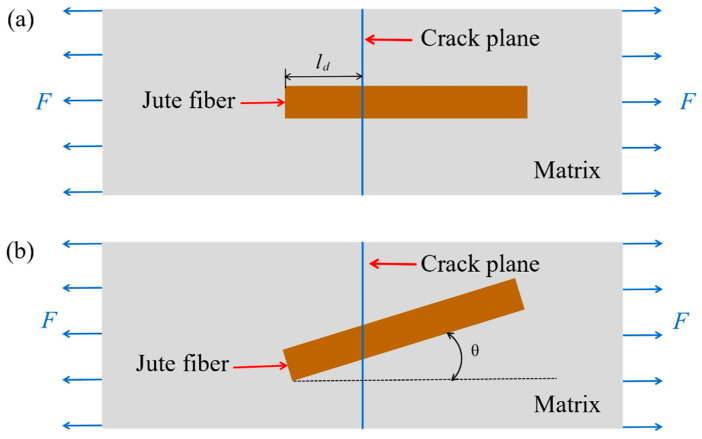
Diagram of the fiber structure passing through the plane of the crack: (**a**) the fibers are parallel to the load loading direction; (**b**) the fibers make an angle *θ* with the normal of the crack plane.

In order to predict the stress required to pull off the short-fiber composite in any direction, the bridging stress of the short fibers in the plane of disruption needs to be calculated [46]. The single-fiber bridging stress is first calculated when the fiber’s major axis direction intersects the crack plane and is in the same direction as the force, as shown in Figure 8a. In Figure 8a, *l_d_* is the shorter section of the fiber, and the bridging stress of the fiber through the crack is

(12) $$\sigma_{f}=\left\{ \begin{array}{ll} 2\tau \frac{l_{d}}{r_{f}} & l_{d}<l_{cf}/2\\ \sigma_{f}^{u} & l_{d}\geq l_{cf}/2 \end{array}\right.$$

When the fibers make an angle *θ* with the normal of the crack plane, as shown in Figure 8b, at this point, the bridging stress of the fiber through the crack is

(13) $$\sigma_{f\theta }=\left\{ \begin{array}{ll} 2\tau \frac{l_{d}}{r_{f}}e^{\mu \theta } & l_{d}<l_{cf\theta }/2\\ \sigma_{f\theta }^{u} & l_{d}\geq l_{cf\theta }/2 \end{array}\right.$$

where *θ* is the angle between the fibers and the normal of the crack plane, and *μ* is the co-efficient of friction between the fibers and the substrate at the intersection point. As shown in Figure 4a, the fiber with spatial coordinates (*θ*, *φ*) intersects the crack plane and the *δ* is the angle between the fiber axial direction (*θ*, *φ*) and the crack plane normal or the load loading direction (*α*, *β*). The relationship between the angles is shown in Equation (1).

At this point, the bridging stress of the fibers through the crack plane is

(14) $$\sigma_{f\delta }=\left\{ \begin{array}{ll} 2\tau \frac{l_{d}}{r_{f}}e^{\mu \delta } & l_{d}<l_{cf\delta }/2\\ \sigma_{f\delta }^{u} & l_{d}\geq l_{cf\delta }/2 \end{array}\right.$$

There is a significant loss in fiber strength due to flexural stresses in the fibers when they cross at an oblique angle normal to the crack plane. The introduction of inclined fiber tensile strength, therefore, helps to predict the tensile strength of short-fiber composites:

(15) $$\sigma_{f\delta }^{u}=\sigma_{f}^{\mu }\left(1-A_{f}\tan\delta \right)$$

where *A_f_* is the composite system constant. The system constant of the short jute/PLA composite is 0.83. When 1 − *A_f_* = 0, i.e., *δ* = 51.34°, the composite is not reinforced by the fibers.

The length of the shorter section of the fiber through the crack plane ranges from 0~*l*/2. When the length of the shorter section of the fiber is less than *l*/2, the short-fiber composite material breaks, and the fiber will completely detach from the matrix under the action of the shear stress *τ*. Otherwise, the fibers will break at the crack surface. Therefore, the average bridging stress is

(16) $${\bar{\sigma }}_{f}=\left\{ \begin{array}{ll} \sigma_{f}^{\mu }\frac{l}{2l_{cf}} & l<l_{cf}\\ \sigma_{f}^{u}\left(1-\frac{l_{cf}}{2l}\right) & l\geq l_{cf} \end{array}\right.$$

When the normal of the fiber and the crack plane forms a spatial angle *δ*, the average fiber bridging stress is

(17) $${\bar{\sigma }}_{f\delta }=\left\{ \begin{array}{ll} \sigma_{f}^{\mu }\frac{l}{2l_{cf}}e^{\mu \delta } & l<l_{cf\delta }\\ \sigma_{f\delta }^{u}\left(1-\frac{l_{cf}}{2l}\right) & l\geq l_{cf\delta } \end{array}\right.$$

From the above discussion, the tensile strength of the short-fiber composite can be derived. Assuming that the total number of fibers in the composite is *M*, *m* is the number of fibers with length *l*~*l* + *dl*, polar angle *θ*~*θ* + *dθ*, and azimuthal angle *φ*~*φ* + *dφ*, and its fiber volume fraction *V_nf_* is

(18) $$v_{nf}=v_{f}\frac{ml}{Ml_{mean}}$$

where *v_f_* is the fiber volume fraction and *l_mean_* is the average length of the fibers. The fiber length and fiber orientation probability density functions *f*(*l*), *g*(*θ*), and *g*(*φ*) were used to correct Equation (18):

(19) $$v_{nf}=v_{f}\frac{l}{l_{mean}}f(l)g(\theta )g(\varphi )dld\theta d\varphi$$

All the fibers contribute to the tensile strength of the composite, which gives the tensile strength of the composite loaded in the (*α*, *β*) direction as

(20) $$\sigma_{c}^{u}(\alpha ,\beta )=\sum_{\varphi =\varphi_{\min}}^{\varphi_{\max}}\sum_{\theta =\theta_{\min}}^{\theta_{\max}}\sum_{l=l_{\min}}^{l_{\max}}{\bar{\sigma }}_{f\delta }v_{nf}+\sigma_{m}v_{m}$$

The integral summation of Equation (20) yields the short-fiber composite tensile strength prediction model, as shown in Equation (21):

(21) $$\sigma_{c}^{u}(\alpha ,\beta )=\int_{\varphi_{\min}}^{\varphi_{\max}}\int_{\theta_{\min}}^{\theta_{\max}}\int_{l_{\max}}^{l_{\max}}f(l)g(\theta )g(\varphi )\frac{l}{l_{mean}}{\bar{\sigma }}_{f\delta }dld\theta d\varphi v_{f}+\sigma_{m}v_{m}$$

The theoretical model of the tensile strength of short jute/PLA composites at any loading angle was obtained by substituting Equation (17) into Equation (21):

(22) $$\begin{array}{ll} \sigma_{c}^{u}(\alpha ,\beta ) & =(\int_{\varphi_{\min}}^{\varphi_{\max}}\int_{\theta_{\min}}^{\theta_{\max}}\int_{l_{\min}}^{l_{cf\delta }}f(l)g(\theta )g(\varphi )\frac{l^{2}}{2l_{cf}l_{mean}}\sigma_{f}^{u}e^{\mu \delta }dld\theta d\varphi \\ & +\int_{\varphi_{\min}}^{\varphi_{\max}}\int_{\theta_{\min}}^{\theta_{\max}}\int_{l_{cf\delta }}^{l_{\max}}f(l)g(\theta )g(\varphi )\frac{l}{l_{mean}}\sigma_{f}^{u}(1-A_{f}\tan\delta )\\ & \times (1-\frac{l_{cf}/(1-A_{f}\tan\delta )}{2le^{\mu \delta }})dld\theta d\varphi )v_{f}+\sigma_{m}v_{m} \end{array}$$

**Figure 9:**
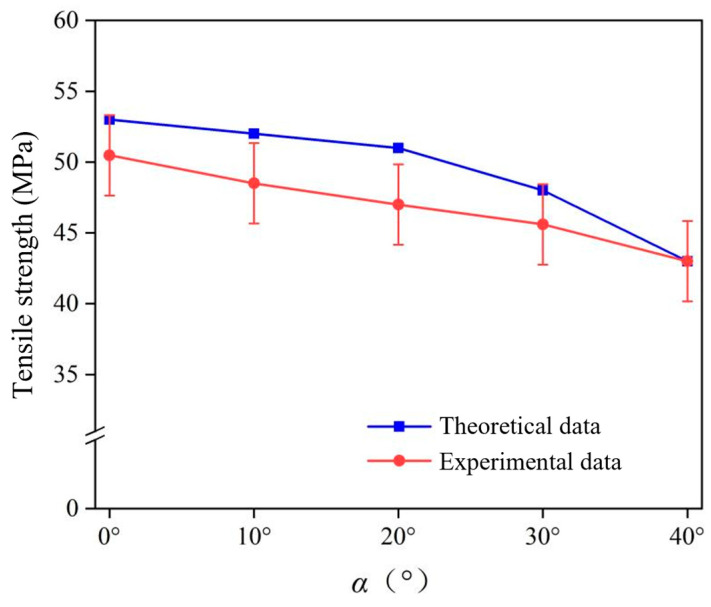
Comparison between tensile strength prediction and experimental results.

Validation of the theoretical model of tensile strength constructed follows. As the maximum angle of *δ* is 51.34°, the maximum value of *α* is obtained by Equation (1) as 40° when *β* is a constant value of 90°. Figure 9 shows the predicted tensile strength of the short jute/PLA composite compared to the experimental results. The experimental and theoretical values of the tensile strength of short jute fibers revealed that the tensile strength of the composite decreases with increasing load loading angle. When the loading angle *α* was 0°, the tensile strength of the composite was 50.8 MPa, and its mechanical properties were lower than those of the laminated composite. When the load loading angle *α* was close to 40°, the tensile strength of the composite was 42.8 MPa, which is lower than the tensile strength of the matrix at 45 MPa. This is due to the unique arrangement of jute fibers in the composite. The maximum relative error between the theoretical and experimental values is 9.2%. These errors are due to the fact that the proposed mechanical prediction model does not take into account the existence of defects at the interface between the fibers and the matrix or the varying diameters of the fibers, which are within acceptable limits, and indicate that the proposed model can predict the tensile strength of short jute/PLA composite at any load loading angle well.

## 4. Conclusions

XCT was successfully used to characterize the fiber orientation distribution (FOD) and fiber length distribution (FLD) in a jute/PLA composite. The probability density functions of the FOD and FLD of the jute/PLA composite can be obtained by quantitative analysis of the XCT data. The laminate theory and the rule of mixtures were modified to obtain strength and modulus prediction models that are applicable to SFRP composites. The results of the predictive models were in good agreement with the experimental measurements. The effect of different loading directions on the mechanical properties of the jute/PLA composite was also investigated. The modulus of elasticity and tensile strength of the jute/PLA composite varied with the loading direction (*α*), and the composite had the maximum modulus of elasticity and tensile strength at *α* = 0°, which gradually decreased with an increase in *α*. This is because the high shear stress of jute fibers in the direction of mold flow made the jute fibers have a unique form of distribution within the composite, resulting in anisotropic mechanical properties of the jute/PLA composite.

## Data Availability

All data are contained within the article.
